# A case report of brief psychotic disorder with catalepsy associated with sequential life-threatening events in a patient with advanced cancer

**DOI:** 10.1186/s13030-017-0095-7

**Published:** 2017-04-11

**Authors:** Mayumi Ishida, Satoshi Kawada, Hideki Onishi

**Affiliations:** 1 Department of Psycho-Oncology, Saitama Medical University International Medical Center, Hidaka City, Saitama 350-1298 Japan; 2 Department of Psychiatry, Okinawa Prefectural Nanbu Medical Center & Children’s Medical Center, Haebaru, Okinawa 901-1193 Japan

**Keywords:** Brief psychotic disorder, Catalepsy, Advanced cancer, Life-threatening event

## Abstract

**Background:**

Cancer is commonly perceived as life-threatening and universally stressful; however, brief psychotic disorder, which occurs in response to extremely stressful events, has not been reported.

**Case presentation:**

A 63-year-old woman, who was diagnosed as having pancreatic cancer with liver metastasis, became unresponsive with very little reaction to verbal contact after sequential life-threatening events, such as thrombosis of both pulmonary arteries and stenosis of the third portion of the duodenum, due to disease progression over 3 weeks beginning with oncological emergency hospital admission. Laboratory findings and electroencephalography were unremarkable. She maintained the position when the psycho-oncologist raised her hand (catalepsy). She had no medical history of psychiatric illness, or alcohol or drug abuse. From these findings, she was suspected of having a brief psychotic disorder with catalepsy and substupor, and 2.5 mg of haloperidol was administered. Her psychiatric symptoms disappeared in 4 days and the diagnosis of brief psychotic disorder was confirmed.

**Conclusions:**

Brief psychotic disorders can manifest in patients with cancer. Careful clinical assessment is needed to correctly diagnose patients with cancer who develop brief psychotic disorders and to identify those who will benefit from correct treatment.

## Background

Cancer is commonly perceived as life-threatening and universally stressful [9, 10], and such perceptions are associated with the pathogenesis of psychiatric disorders and physical illness. The prevalence of psychiatric disorders is high among patients with cancer during both treatment and the terminal stages [3, 7]. The rates of death due to cardiovascular diseases and suicide after receiving cancer diagnosis are also high compared with the general population [5].

Brief psychotic disorders include those that occur in response to extremely stressful events, which are defined as lasting from 1 day to 1 month followed by a full return to premorbid levels of functioning [1]. However, no patients with cancer who developed brief psychotic disorder have been reported to date.

Here, we describe a patient who developed a brief psychotic disorder after being diagnosed with advanced pancreatic cancer and then experienced sequential life-threatening events soon thereafter.

## Case presentation

A 63-year-old woman with advanced pancreatic cancer was referred for a psycho-oncology consultation due to her response, which seemed like sudden onset of loss of consciousness.

On visiting her, we found her bedridden, staring at the ceiling without blinking, and responding minimally but correctly to our questions, including orientation-related ones such as date and location. Her family said that she suddenly became absentminded and stated to her husband, “It is over and I will probably die soon.”

Her clinical findings were as follows: blood pressure, 109/70 mmHg; heart rate, 78 beats/min; oxygen saturation, 98%; body temperature, 36.7 °C. Laboratory findings revealed that alkaline phosphatase and amylase values were slightly increased, but these could not explain her psychiatric symptoms. By electroencephalography, the basic rhythm was 8–10 Hz and slow waves were not evident, findings that did not suggest reduced consciousness. Neurological examination demonstrated that she maintained the position when the psycho-oncologist raised her hand (catalepsy).

She had no medical history of psychiatric illness or alcohol or drug abuse, and she was very kind to others. Her husband said that she was very shocked at her diagnosis of pancreatic cancer, and became anxious about treatment and admission to hospital. She had been afraid of cancer because her grandfather had died of cancer after a long period of suffering.

She had been diagnosed with advanced pancreatic cancer accompanied by multiple liver metastasis 4 months before the event, and gemcitabine was administered. Three weeks before the event she had been admitted to our emergency department with thrombosis of both pulmonary arteries and stenosis of the third portion of the duodenum due to disease progression. She frequently vomited. Insomnia and anxiety reappeared, and after much agonizing, she decided to undergo a gastrointestinal bypass. The surgery proceeded 3 days before the event, without complication and post-operative delirium was not evident.

Based on physical, neurological, and psychiatric assessment, we suspected brief psychotic disorder with catalepsy and substupor, and she was administered 2.5 mg of intravenous haloperidol.

On the following day, she could sit on the bed and respond to our questions more clearly. Two days later, she was able to make expressions and smiled at us. The symptoms disappeared 4 days later and did not reappear. One week later, she told us about this experience and said that it was very scary. We diagnosed a brief psychotic disorder based on these findings, and its features fulfilled the Diagnostic and Statistical Manual of Mental Disorders 5th edition [1].

## Discussion and Conclusions

We described a patient with cancer who developed a brief psychotic disorder that was correctly diagnosed after a detailed clinical examination. We believe that being diagnosed with unresectable pancreatic cancer and soon after experiencing a life-threatening pulmonary artery embolism and small intestinal obstruction were related to the pathogenesis of the disorder.

Correct diagnosis is very important because a misdiagnosis could result in a change in treatment and irrelevant medication being administered, which would increase the possibility of drug-related adverse events [6, 11].

Several differential diagnoses were considered for this patient. Delirium was considered because of her poor response. This frequently develops after surgery [4] and in patients with advanced cancer [8]. However, we could not find any indicators of delirium; catalepsy is not considered as a symptom of delirium. Furthermore, the basic EEG rhythm ruled out delirium.

Another differential diagnosis was schizophrenia because the patient developed catatonia, which sometimes occurs in patients with schizophrenia [2]. However, she did not have a history of schizophrenia and did not develop any other symptoms that are characteristic of this condition.

Although cancer is commonly perceived as a life-threatening, universally stressful event [9, 10], associations with brief psychotic disorders have not been reported to date. We believe that this disorder is misdiagnosed or underestimated. Detailed clinical observation is likely to uncover new patients.

In conclusion, brief psychotic disorders can manifest in patients with cancer. Careful clinical assessment is needed to correctly diagnose patients with cancer who develop brief psychotic disorders and to identify those who will benefit from correct treatment.
